# Interaction between Oligodendrocytes and Interneurons in Brain Development and Related Neuropsychiatric Disorders

**DOI:** 10.3390/ijms25073620

**Published:** 2024-03-23

**Authors:** Yingqi Liu, Jie Yuan, Yuhao Dong, Sufang Jiang, Ming Zhang, Xianghui Zhao

**Affiliations:** Department of Neuroscience, Air Force Medical University, Xi’an 710032, China

**Keywords:** oligodendrocyte, interneuron, brain disorders, neurodevelopment

## Abstract

A variety of neurological and psychiatric disorders have recently been shown to be highly associated with the abnormal development and function of oligodendrocytes (OLs) and interneurons. OLs are the myelin-forming cells in the central nervous system (CNS), while interneurons are important neural types gating the function of excitatory neurons. These two types of cells are of great significance for the establishment and function of neural circuits, and they share similar developmental origins and transcriptional architectures, and interact with each other in multiple ways during development. In this review, we compare the similarities and differences in these two cell types, providing an important reference and further revealing the pathogenesis of related brain disorders.

## 1. Introduction

Oligodendrocytes (OLs) are the myelin-forming cells in the central nervous system (CNS). The myelin sheath is important for the rapid saltatory conduction of nerve impulses along axons and maintaining normal communications between neurons. Defects in the myelin sheaths of OLs caused by developmental and pathological injuries lead to various neurological and psychotic disorders, including cognitive disorders, anxiety, and depression [1]. On the other hand, GABAergic inhibitory interneurons are important neural types regulating the output of excitatory neurons, and are key to maintaining an excitation/inhibition balance in neural microcircuits. These cells are thought to be one of the key causes of numerous psychotic disorders [2]. To comprehend the interaction between these two cells in the development of the brain and related disorders, we compare and review the developmental origins, transcriptional regulation and interactive modes of these two neural cell types. This review will provide a comprehensive understanding for researchers working on developmental brain diseases and neuron–glia interactions.

## 2. The Origins of Oligodendrocytes

**Figure 1:**
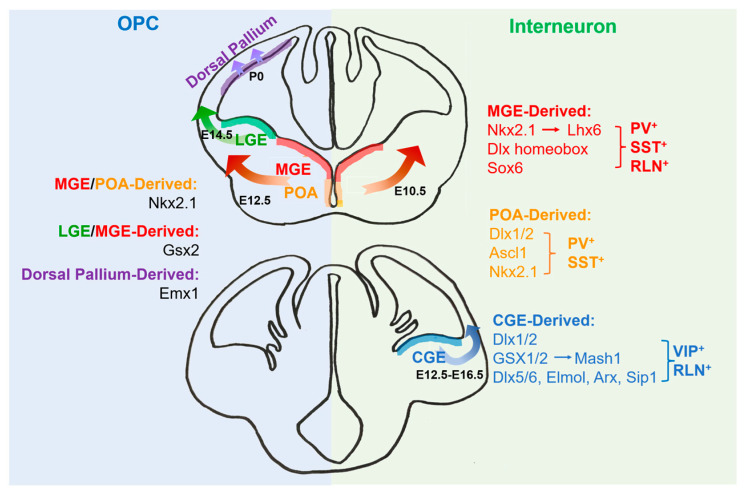
Schematic diagram shows the generation and transcriptional regulation of OPCs and interneurons from the embryonic telencephalon. OPCs originate from three regions: the ganglionic eminence, the preoptic area (POA) and the dorsal pallium. The regions generating cortical and hippocampal interneurons are the medial ganglionic eminence (MGE), the caudal ganglionic eminence (CGE) and the POA. A distinct combination of transcription factors is required for the specification of these two cell types, as indicated.

In the development of the rodent forebrain, OLs are differentiated from oligodendrocyte precursor cells (OPCs) in the ganglionic eminence (GE) and the cortex ventricular zone at different stages [3], and there are three continuous OPC-generation waves (Figure 1). The first wave starts around embryonic day 12.5 (E12.5) in the mice, from precursor cells expressing the transcription factor NKX2.1 in the medial ganglionic eminence (MGE) and embryonic preoptic area (ePOA); the second wave starts at E14.5, from the precursor cells expressing the homeobox gene *Gsx2* in the lateral ganglionic eminence (LGE) and the MGE; and the third wave starts after birth from precursor cells expressing the homeobox gene *Emx1* in the dorsal pallium [3]. The Cre-loxP-lineage-tracing study indicates that the fate-specification of three OPC-generation waves are as follows: 10 days after birth in the cortex, the first-wave OPCs from the MGE and ePOA are eliminated and replaced by the second- and third-wave OPCs. Although the first-wave OPCs still survive in other brain regions, their deletion in the neocortex suggests this cluster may have little effect on cortical circuit formation and axonal myelination [4]. Moreover, removing the first-wave OPCs through gene knockout confirms that the replacement of this cell cluster does not cause significant changes in the myelin sheath, suggesting the functional redundancy of these cells compared with other OPCs [4].

In the outer subventricular zone (oSVZ) of primate brains, there exists a special radial glia subtype, the outer radial glia (oRG), which is believed to be beneficial for the expansion of gray matter [5]. The single-cell sequencing analysis of the human brain demonstrates that there exist pre-OPCs, originating from the local intermediate progenitor cells [5]. Pre-OPCs express oRG-specific genes, such as *PTPRZ1*, *TNC*, *MOXD1*, *HOPX* and *FAM107A* [5]. This indicates that these cells may be produced from oRGs. Furthermore, in the early developmental phase of human glial cells (GW20–24), there are numerous epidermal growth-factor receptor/OL transcription-factor 2 double-positive (EGFR^+^OLIG2^+^) cells in the oSVZ of the cortex germinal zone. These progenitor cells are present in low abundance during GW16–18, but then increase dramatically during GW20–24, consistent with the generation time of OPCs. Different from the oRG with long radial process, cells in the oSVZ expressing EGFR distribute randomly in the germinal zone; this distribution could be vertical, horizontal or oblique to the surface [5]. Most EGFR^+^OLIG2^+^ cells do not express pS6, an oRG marker and a canonical readout of activated mTOR signaling [6]. In contrast, most TNC^+^SOX2^+^ oRGs in oSVZ do not express EGFRs [7]. These findings support that EGFR labels a population of progenitor cells in oSVZ distinct from oRGs. Moreover, EGFR^+^OLIG2^+^ cells are EOMES^−^ and PPP1R17^−^, suggesting a lineage separation between OLs and neurons. Together, these findings demonstrate that EGFR expression marks pre-OPCs in the human oSVZ. These progenitor cells can produce OPCs locally in the second and third trimesters. The quantity of OPCs increases greatly through long-term proliferation and symmetric division in the subsequent developmental process, laying a foundation for the later generation of OLs [5].

## 3. The Origins of Interneurons

Like the origins of OLs, cortical interneurons are mainly generated from the MGE and the caudal ganglionic eminence (CGE). In the mouse brain, the MGE is believed to be the origin zone of 50–60% of cortical interneurons, which appear before the generation of the first wave of OPCs, at around E10.5 [8]. Meanwhile, the CGE produces 30–40% of cortical interneurons, which arise between E12.5 and E16.5 [8]. In mammals, GABAergic interneurons first begin tangential migration at E12.5, which is the time point of first-wave OPC generation [9]. After arriving in the cortex, early interneurons migrate horizontally in cortical plates and then more interneurons are produced, migrating through the intermediate zone. At a later time of cortical formation (E14–15), three migratory routes (also known as tangential migration flows) can be observed in the cortex, from the marginal area, basal area and lower middle and subventricular area [10].

Progenitor cells in the MGE can produce both GABAergic interneurons and OPCs, indicating the same progenitor for these two cell types. At present, it is known that interneurons generated from the MGE and CGE are complementary subtypes that have strong corresponding relationships with their source-specific progenitor cells. For example, the MGE mainly generates parvalbumin (PV) interneurons (including basket cells and chandelier cells) and somatostatin (SST) interneurons, among which the largest subset is formed by Martinotti cells [11]. Meanwhile, the CGE produces relatively rare subtypes, including bipolar and vasoactive intestinal peptide (VIP)-expressing multipolar interneurons [12]. Although hippocampal genetic lineage analysis has emphasized that specific fetal brain structures produce specific interneuron subtypes [12], there is not a simple corresponding relationship between the germinal zones and interneuron subtypes. For instance, in different cerebrum zones, several interneuron types (such as fast-spiking basket cells) display apparent similarity in histologic origins, but other types do not seem to. Additionally, a special type of interneurons called orientation lacunar molecular cells have at least two origins and produce different subclasses, e.g., ionic serotonin receptor 5HT3aR-positive and -negative cells. More interestingly, the main source of interneurons in the basal ganglia is the MGE [12].

## 4. Transcriptional Regulation for the Development of Oligodendrocytes and Interneurons

Neural stem cells (NSCs) are the cell source of the CNS. They express basic helix–loop–helix (bHLH) and NK homeobox factors when differentiating into OPCs. These transcription factors play an important role in the specification of OPCs, including ASCl1/MASH1, OLIG1 (OL transcription factor 1) and OLIG2, as well as NKX2.2, NKX6.1 and NKX6.2 [13,14,15] (Figure 1). For instance, OLIG2 inhibits the generation of astrocytes from NSCs by repressing nuclear factor IA (NFIA) [16]. SRY-box (SOX) family members also take part in different stages of OPC formation, differentiation and maturation. In the early stage, SOX1, SOX2 and SOX3 keep OPCs in the undifferentiated state [17]; SOX11 and ETS relative gene 1 are regulated by histone deacetylases (HDACs) and inhibit the expression of myelin genes. SOX10 is another important factor controlling OPC differentiation, and SOX10 expression may be regulated by OLIG2 via a distant evolutionarily conserved enhancer of the SOX10 gene and a promoter-dependent mechanism [18]. A recent study shows that the phosphorylation of FOXO1 via AKT, a serine/threonine (Ser/Thr) kinase, is important for SOX10 expression and OL differentiation in vitro [19]. Analyses of various animal models have revealed an essential role of SOX10 in the terminal differentiation of OLs in coordination with OLIG1, MYRF (myelin regulatory factor) and TCF4 (transcription factor 4) [20]. MYRF is identified as a decisive factor that helps SOX10 to switch between its target genes during the OL-differentiation process [21]. Moreover, Ying Yang 1 (YY1) acts as a lineage-specific repressor of transcription inhibitors of myelin gene expression (TCF4 and ID4) by recruiting HDAC1 to their promoters during OL differentiation [22].

Several transcription factors, such as LHX6, SOX6, NKX2.1 and the DLX homeobox gene, play important roles in regulating the generation of PV and SST interneurons from the MGE [23,24]. For instance, the expression of NKX2.1 is limited in the MGE, and the straight or conditional knockout of *Nkx2.1* leads to decreased pools of both PV and SST interneurons. LHX6 is one of the targets of NKX2.1, and its expression is also limited to the MGE [25]. In *Lhx6* deficiency, neural progenitors generated from the MGE can still migrate to the cortex correctly; most of these neurons do not express PV or SST, but their expression of neuropeptide Y increases. However, in the *Lhx* mutant, PV and SST interneurons are not eliminated completely, which indicates that the MGE is not the only origin of these two interneuron types [23] and suggests there are transcription factors other than NKX2.1 and LHX6 regulating the differentiation of PV and SST interneurons [25]. DLX1 and DLX2 show redundant effects in the formation of interneurons. Mice with a single mutation of *Dlx1* or *Dlx2* exhibit minor defects in the formation of GABAergic neurons; however, double knockouts of *Dlx1* and *Dlx2* lead to a global defect in the development of GABAergic neurons, including the achievement of GABAergic characteristics, the initiation and termination of tangential migration and the functional maturation of certain subclasses [26]. Meanwhile, transcription factors like ELMO1, DLX5/6, ARX and SIP1 have all proven necessary for the migration and specification of certain interneuron subtypes [23,27]. A mutation in these genes may lead to abnormal neural circuits and produce psychosis in mice [12]. In addition, GSX1 and GSX2 homeobox proteins are involved in the differentiation of interneurons generated from the CGE. Mutants in *Mash1*, a downstream target gene of GSX1/2, show a significant decrease in cortical interneurons in early development [28]. Interneurons generated from the POA, expressing DLX1/2, ASCL1 and NKX2.1, contribute only 10% of the total population in the adult mice cortex but include a large diversity of subtypes, such as SST^+^, PV^+^ and RLN^+^ [29].

OLs and interneurons share similar transcriptional architectures during early brain development since they are both generated from the GE. For instance, DLX homeobox proteins inhibit the formation of OPCs by acting on their common progenitor cells [27]. In newborn wild-type mice, transplanted progenitor cells from the ventral telencephalon of the *Dlx1/2* mutant can differentiate into OLs and survive to form a myelin sheath in the adult. These studies confirm the essential role of DLX in the regulations of interneuron and OL specifications, especially in the ventral forebrain of the embryonic stage [27].

Recent studies revealed that DLX1/2 can negatively regulate the formation of OPCs by interacting with numerous partners. For example, MASH1 is relevant to the regulation of neurons in the telencephalon and the formation of OLs, whose function is necessary for the development of olfactory bulb neurons and perinatal OLs, and it can promote the formation of OPCs by limiting the number of DLX^+^ progenitor cells [30]. For one, MASH1 could combine with DNA regulatory components between DLX1 and DLX2. Furthermore, the expression of MASH1 increases in the cortical ventricular zone and subventricular zone of the mutants of *Dlx1* and *Dlx2* [30]. Some findings confirm that in the germinal area of MGE and AEP, the combined expressions of transcription factors DLX1/2, OLIG2 and MASH1 inhibit the formation of OPCs in the ventral forebrain by regulating the balance between the formation of forebrain neurons and OPCs; namely, DLX1/2 inhibits the formation of OPCs in the ventral forebrain by negatively regulating the expression of OLIG2. The presented studies suggest that before birth, once it is decided that neural progenitor cells will become OL-lineage cells, they continue to express OLIG2 and inhibit the interneuron transcription factor DLX2.

In addition, transcription-factor patterns, including GSX2 and DLX1/2, are required to specify interneurons and repress oligodendroglial fate. After birth, the niche of GSX2^+^ neural stem cells is derived from its GE counterpart, regulated by a similar transcription factor hierarchy, which persists in the subventricular zone (SVZ) of the murine brain [31].

Interestingly, there exists a fate switch between the two cell types. An overexpression of DLX2 alone in postnatal mouse OPCs switches their lineage fate to GABAergic neurons within two days by downregulating OLIG2 and upregulating a network of inhibitory neuron transcripts [32]. Functionally, inhibitory neurons generated from the trans-differentiated OPCs can create an action potential and form GABAergic synapses. This study suggests that the molecular regulatory network shared by the two types of cells plays an important role in promoting the fatal switch from OPCs to interneurons in specific situations during the developmental process, such as developmental abnormalities and disease.

## 5. The Interaction between Interneurons and Oligodendrocytes

**Figure 2:**
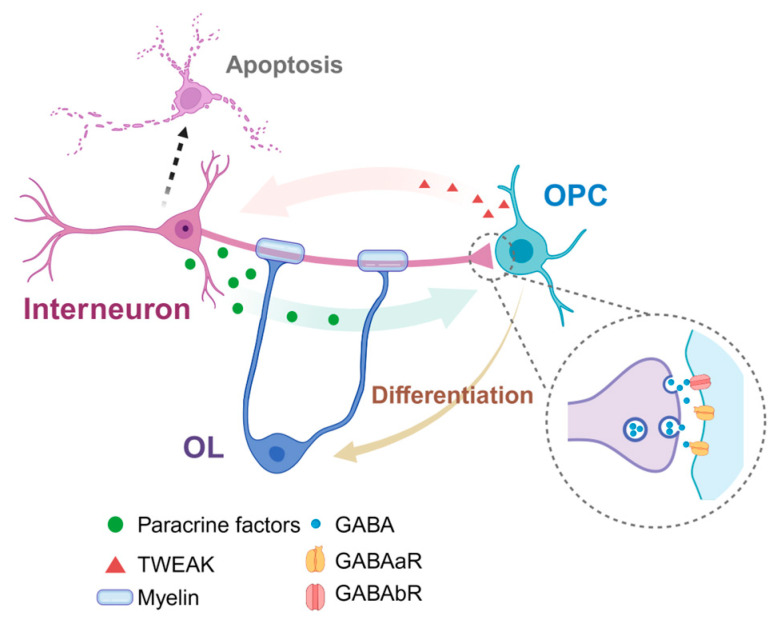
Schematic diagram shows the crosstalk between oligodendrocyte and interneuron.

From the above introduction, we can identify several common characteristics between GABAergic interneurons and OLs in the developing cortex: ➀ these two kinds of cells are generated from the same germinal area, and their precursor cells use the same group of transcription factors; ➁ they both migrate to the cortex along similar tangential routes [33]; and ➂ they are both over-produced early after birth and their numbers then significantly decrease [33]. Moreover, there exist plenty of interaction modes between the two cells during brain development. For instance, migrating interneurons can release paracrine factors and promote the differentiation of OPCs [34]; OPCs in the cortex could accept temporary and primary synapse inputs from PV interneurons before the peak of differentiation; and most PV interneurons in the cortex are myelinated [33]. Research in recent years has discovered that non-synaptic modes of interneuron-to-oligodendroglia communication, such as extrasynaptic transmission or mechanical interaction, will influence oligodendroglia function and myelination. We will elucidate their interactions in the following sections (Figure 2).

### 5.1. The Synaptic Contact between Interneurons and Oligodendrocytes

At present, OPCs are the only glial cells that are known to receive direct inputs from both the GLU^+^ excitatory neurons and GABA^+^ inhibitory neurons [35]. The synapses between OPCs and glutamatergic neurons can be found in numerous brain areas, including the hippocampus, cerebellum, cortex, brain stem and white-matter bundles, hinting that receiving a synaptic signal is an important and common characteristic of OPCs [36]. In 2004, LIN et al. first detected interneuron–OPC synapses in acute hippocampus slices [37]. Interneurons in the CA1 area directly release GABA, which acts on the postsynaptic GABA_A_ receptors in OPCs and is the prime inhibitory neurotransmitter in the CNS. More evidence further proves the existence of these inhibitory neuron–OPC synaptic connections [35,38]. In the cortex, the inhibitory synapses account for around 90% of all synapses received by OPCs [38]. Such synaptic transmissions through GABA_A_ receptors peak at two weeks after birth, with an immediate increase in the number of OLs. Until four weeks after birth, their communication mode changes into a non-synaptic mode, when the GABAergic electric currents in OPCs are mainly generated by GABA overflowing. It is worth noting that OPCs in the cortex have basically finished differentiation at this age. These results further indicate that the early postnatal establishment of OPC–interneuron synapses in the cortex is necessary for OPC differentiation and interneuron myelination [39].

Furthermore, the first wave of OPCs in the cortex generate functional clusters with their lineage-relative interneurons. Due to their common developmental origins, these two clusters form synaptic connections in priority, which are maintained until the formation of mature oligodendrocytes. In the sensory cortex of mice, the number of OPC–interneuron synaptic connections peaks at ten days after birth and then decreases, reflecting an accurate and transient time window. Interestingly, this happens during the extensive programmed death of both interneurons and OPCs in the developing cortex. Actually, in the first two weeks after birth, the first wave of generated OPCs and 40% of interneurons are eliminated. This cell death and the highly regulated transient synaptic connections together indicate that the two kinds of cells are highly dynamic during development [4].

Although neurons can both receive and emit signals in the neural circuit, OPCs seem to only receive inputs as postsynaptic components [40]. The synapses between glutamatergic axons and OPCs are built early during the development and become stronger with age (greater currents and more inputs), which is in parallel with the normal development of peripheral neuron synapses and different from the GABAergic signal in OPCs as mentioned above.

### 5.2. Effects of GABA Signal in Differentiation and Myelination of OPCs

GABA can bind to both GABA_A_ or GABA_B_ receptors, exerting fast or slow inhibition. In brain slices containing the corpus callosum and hippocampus, the GABA_A_ receptor has been found to induce the depolarization of OPCs, which is probably relative to cell differentiation [39]. The expression of GABA_A_ receptors decreases during the differentiation from proliferative OPCs to myelinating OLs. Recently, transcriptome studies and single-cell real-time quantitative PCR assays indicate that all GABA_A_-receptor subunits (α1–5, β1–3 and γ1–3) decrease during the development process of OLs [41,42,43]. The γ2 subunit is expressed in OPCs but not in OLs [41,42,43]. However, a deficiency of the γ2 subunit does not seem to impact the proliferation and differentiation of OPCs. It is interesting that while the number of OPCs expressing the α2, α5, β1 and γ2 subunits decrease 2–4 weeks after birth, those expressing the α3 and α4 subunits increase; this is consistent with the time window of transforming synaptic transmission to non-synaptic communication. The γ2 subunit is specially detected on the postsynaptic membranes of PV interneuron–OPCs, whose expression levels are comparable to that in neurons [38]. Thus, the γ2 subunit is necessary for the postsynaptic clustering of the GABA_A_-receptor subunit in OPCs.

It is known that the GABA signal plays a key role in the origins of OPCs, as well as in axon identification and myelination [35,44]. The systemic administration of the GABA_A_-receptor antagonist bicuculline can significantly induce the proliferation of OPCs, while increasing the level of GABA induces the opposite effect in cerebellar white matter [35]. In mice brain-slice cultures, endogenous GABA produces an equal number of OPCs and mature OLs, which can be reversed by the GABA_A_-receptor antagonist gabazine [45], indicating that the GABA_A_-receptor signal pathway inhibits the self-renewal and myelination of OPCs.

Furthermore, the GABA signal also takes part in regulating OL functions in brain diseases involving demyelination. Studies on brain stroke have discovered that the release of GABA increases rapidly in the ischemic penumbra [46]. However, GABA_A_-receptor-mediated inputs on OPCs decrease [35], alongside more proliferating OPCs and a delayed maturation of OLs. With the stimulation of GABA, OPCs in the human cortex produce neurotrophins such as BDNF, which increase after brain stroke [47]. BDNF can also promote the proliferation of OPCs in both physiological and pathological situations [48]. It is still unclear whether these newly generated OPCs take part in the regeneration process.

Both postsynaptic and presynaptic GABAergic transduction decrease in the brains of patients diagnosed with progressive demyelinated multiple sclerosis (MS) [49]. The GABA level in the sensory motor cortex increases, while that in the hippocampus decreases. In experimental autoimmune encephalomyelitis (EAE) mice, an animal model for MS, a single-cell RNA-seq study revealed that the level of the GABA_B1_ subunit decreases in mature oligodendrocytes, and the levels of the GABA_B2_ and GABA_A_ receptors remain unchanged [50]. This indicates that the GABA_B_ receptor may be involved in the remyelination process after damage, the same as under normal physiological conditions. Interestingly, the expression of GABA transporter GAT3, responsible for exporting GABA out of cells, decreases in OPCs, and the level of GAT1 rises in these EAE mice [50]. It is difficult to judge whether the change in GAT expression is the result of or reason for demyelination. And it remains to be clarified how the GABAergic signal in oligodendrocyte-lineage cells participates in demyelination and remyelination.

### 5.3. Density and Myelination of GABA^+^ Interneurons

Oligodendrocytes have been regarded as an indispensable regulator of fast-spiking PV^+^ interneuron density and myelination [51]. At the first two postnatal weeks, OPCs sense interneuron activity through the expression of GABA_B_ receptors (GABA_B_R) and concomitantly adjust the interneuron density by releasing a TNF-like weak inducer of apoptosis (TWEAK) [52]. The conditional knockout (cKO) of *Gababr* in OPCs interrupts interneuron–OPC signaling through an attenuated release of TWEAK and results in increasing the population of PV^+^ interneurons. However, these interneurons appear hypoactive, with fewer contacts with OPCs during development, and exhibit deteriorated myelin structures in adulthood. Patch-clamp recordings reveal reduced interneuron activity, and EEG recordings also detect impaired cortical network activity in mutant mice [52]; both suggest an imbalance of excitation and inhibition (E/I) in the mutant mPFC.

According to the morphological analysis of the murine cortex, half of the myelin sheaths from layers 2 and 3 and 25% of the myelin sheaths from layer 4 are all wrapped on inhibitory neurons, especially on PV^+^ basket cells [53]. Axons of PV interneurons make contact with neuronal cell bodies (especially pyramidal neurons) and their proximal dendrites. Other types of interneurons in the cortex (such as VIP^+^ and SST^+^ interneurons) have a much lower extent of myelination. Recent studies confirm that the lack of the γ2 subunit of the GABA_A_ receptor in OPCs may interrupt the interaction between PV interneurons and OPCs, leading to a lower level of myelination in PV interneurons from the barrel cortex [54]. Although PV interneurons form synaptic connections with OPCs in priority, the connections do not seem to be necessary to initiate the myelination process on PV interneurons. The possible reasons for this are as follows: There also exist synaptic connections between unmyelinated PV interneurons and OPCs, and PV interneurons preserve the myelin structure when synapses between PV interneurons and OPCs are deactivated. In the functional cell clusters consisting of survived OPCs and lineage-relative interneurons during development, the first-wave OPCs that start to differentiate to myelinating OLs are irrelative to the neuronal characteristics, and axons of both glutamatergic and GABAergic interneurons are myelinated. Promoting the survival of interneurons and OPCs derived from the first wave will lead to a significant expansion in other OPC populations, so the whole myelination level would be elevated [4].

It is worth noting that GABA signaling may participate in the regulation of myelin formation. In fact, the increase in synaptic connections between interneurons and OPCs is beneficial for the myelination of deep-layer neurons in the sensory cortex [4]. Moreover, the myelination process mediated by GABA signaling may be different from the glutamic-acid-directed process: compared to non-GABAergic neuronal axons, the distances between the nodes of Ranvier are shorter and the protein level of the myelin basic protein is higher in GABAergic neurons [53].

## 6. Neuropsychiatric Disorders with Interneuron and Oligodendrocyte Involvement

### 6.1. Schizophrenia

Schizophrenia (SCZ) is a serious mental disorder in which people interpret reality abnormally. Impaired cognitive ability is considered a core feature of this disorder. Dysmyelination and interneuron defects during adolescent prefrontal cortex (PFC) development have been hypothesized to be the causes of SCZ [55,56,57,58], and these may contribute to the observed cognitive disturbances in individuals affected by this disorder [56,59,60]. In our previous observation, we identified the essential role of DNA hydroxymethylation in OPC differentiation and myelination, and further revealed two core endophenotypes of SCZ, impaired working memory and sensorimotor gating ability, in dioxygenase deficient mice [61]. Moreover, it seems that only specific cortical interneuron classes are affected in schizophrenia: PV^+^ interneurons in the dorsolateral PFC of adult patients with SCZ demonstrate a decreased expression of GAD67 [62], hinting that these cells are less able to inhibit pyramidal neurons. Since PV^+^ interneurons include basket and chandelier cells, their defects may reflect a perturbation of pyramidal neurons in both perisomatic and axo-axonic inhibition. Furthermore, electroencephalogram synchronization in the gamma range is impaired in the PFC of patients with schizophrenia when performing working-memory tasks [59] which mainly originates from these fast-spiking PV interneurons.

Three possible mechanisms have been suggested for how PV^+^ interneurons contribute to SCZ [60]. First, there may be a defective inhibitory transmission from interneurons onto cortical pyramidal cells in individuals with SCZ [63]. Second, the defects in potent excitatory drives from pyramidal cells cause failure in recruiting PV^+^ interneurons, thus impairing the inhibitory function of PV^+^ interneurons. Finally, numerous research works show that interneurons’ impaired excitation is present in both mouse models of schizophrenia and in patients with the disease [64]. Therefore, a decreased excitatory drive to PV^+^ interneurons consistently leads to a reduced expression of GAD67 mRNA [65], which is believed to cause a decreased activity of these interneurons in turn.

The impaired bidirectional OPC–interneuron signaling induces defective social cognitive behaviors in the *Gababr* cKO mice mentioned above [52], indicating that their interactions might be a good candidate for potential therapeutic interventions for SCZ. The evidence that supports the involvement of OL and interneuron dysfunctions in SCZ is summarized in Table 1. 

### 6.2. Depression

Depression is another common mental disorder, affecting more than 300 million people worldwide. Both OLs and interneurons have a shown strong association with the pathology of depression. Oligodendrogenesis and myelination in the PFC are highly sensitive to stressful experiences, including physiological and pathological conditions. Liu et al. reported that protracted social isolation decreases myelin gene expression and nuclear heterochromatin formation, thus inducing transcriptional and ultrastructural changes in OLs of the PFC, which ultimately leads to impaired adult myelin formation [66]. Indeed, in stress-induced depressive states in mice, 69% of the most significantly downregulated genes were myelin-related, such as myelin oligodendrocyte glycoprotein and ermin [67]. Moreover, the anti-depressant venlafaxine, a serotonin- and norepinephrine-reuptake inhibitor, successfully improved cognitive impairment and depression-like behaviors in a cuprizone-induced demyelinated mouse model [68]. In our recent research, we revealed novel insights for calcium homeostasis in manipulating developmental transition from OPCs to pre-OLs [69]. We also showed that the loss of the endoplasmic reticulum calcium channel ITPR2 in OLs induces anxiety-/depressive-like behaviors in the mice.

In the cortex, hippocampus and amygdala of major-depressive-disorder suicide victims, a microarray revealed significant reductions in quaking gene transcripts (QKI), which is a highly conserved RNA-binding protein and is important for myelination in the postnatal CNS [73]. Since the QKI-6 isoform interacts with argonaute 2 and MBP mRNA in cytoplasmic granules of OLs during cellular stress [74], QKI may play a specific role in myelination-related deficits in the etiology of psychiatric disorders [75]. Moreover, the microarray analysis of postmortem tissue from depressive individuals demonstrates that myelination or OL-lineage-related gene transcripts are remarkably downregulated with worsened symptoms [76,77]. In agreement with these observations, an animal model of chronic unpredictable mild stress showed a decreased expression of OL-associated genes, such as MBP, MOB and CNP [70]. Further phenotype-based genetic-association studies suggested that the CNP SNP rs2070106 AA genotype influences myelin/axon integrity in the frontal corpus callosum fibers from SCZ patients and that CNP rs2070106 might be causative of a catatonic depression syndrome with age [71].

In addition, GABAergic interneurons generated from pluripotent stem cells (iPSCs) from major-depressive-disorder patients with suicidal behaviors (sMDD) exhibit increased neurite arborization, increased neural firing and decreased calcium-signaling propagation [72], which could be the early pathology of sMDD. Similarly, increased neural morphology is discovered in MDD serotonergic interneurons. According to a transcriptomic sequencing study, the reduced expression of serotoninergic receptor 2C (5-HT2C) in GABAergic interneurons may lead to defective neuronal activity in patients with sMDD, which may temporally delay the release of intracellular calcium [78]. Targeting the 5-HT2C receptor through agonist or genetic approaches can restore neuronal activity deficits in sMDD GABAergic interneurons [72]. Together, these observations suggest the strong implication of oligodendrocytes and interneurons in depression.

## 7. Conclusions

Recent studies based on single-cell sequencing indicate that both OLs and interneurons are highly heterogeneous, consisting of varieties of subtypes with specific gene-expression profiles and functions [79]. However, how these complicated cell subtypes interact in a coordinated manner to regulate neural microcircuits has not yet been well defined. Brain mesoscopic atlas imaging, neural modulating technologies, multi-omics sequencing and conjoint analysis will provide strong support to reveal the cellular and molecular phenotypes of neurological and psychiatric disorders, involving the deficiency of OLs and interneurons. In addition, researchers should pay attention to the species-level differences in origin and lineage specialization between the two kinds of cells in rodents and humans, which may help in the design of reliable strategies for brain-disease treatment. In conclusion, further studies elucidating novel interactions between these two cell types under physiological and pathological conditions may provide important instructions for exploring new therapeutic targets and strategies for related diseases.

## Figures and Tables

**Table 1:** Representative evidence of interneuron and oligodendrocyte involvement in schizophrenia and depression.

Neuropsychiatric Disorders	Evidence of Interneuron or Oligodendrocyte Involvement	Reference
Schizophrenia	Dysmyelination during adolescent PFC development in mice model	[55,57,58,61]
	Decreased expression of GAD67 in PV+ interneurons of SCZ patients	[62]
	Conditional knockout of *Gababr* in OPCs interrupting the impaired bidirectional OPC–interneuron signaling and induing defective social cognitive behavior in the mice	[52]
	Impaired electroencephalogram synchronization in the gamma range, originated mainly from PV+ interneurons, in SCZ patients	[59]
	Fewer inhibitory synapses from interneurons onto cortical pyramidal cells in SCZ individuals	[63]
	Defects in potent excitatory drives from pyramidal cells, causing the failure in recruiting PV^+^ interneurons in mice models	[65]
	Impaired excitation of interneurons in both mouse models and SCZ patients	[65]
	Decreased excitatory drive to PV^+^ interneurons, leading to a reduced expression of GAD67 mRNA	[66]
Depression	Decreased myelin gene expression and impaired myelin formation in protracted social-isolation mice	[66]
	Myelin genes are the most significantly downregulated in stress-induced depressive mice	[67]
	Cuprizone-induced demyelinating mice develop depression-like behaviors	[68]
	Calcium homeostasis in OLs is essential for myelination and causes anxiety/depressive like behaviors once interrupted in OLs	[69]
	Animal models of chronic unpredictable mild stress show a decreased expression of OL-associated genes	[70]
	Myelin gene mutation might be a causative of catatonia-depression syndrome in patients	[71]
	Interneurons exhibit abnormal morphology and function in sMDD patients	[72]
	Targeting the 5-HT2C receptor can restore neuronal activity deficits in sMDD GABAergic interneurons	[72]
